# (*E*)-2-(4-Methylbenzylidene)hydrazinecarboxamide

**DOI:** 10.1107/S1600536810052797

**Published:** 2011-01-08

**Authors:** Yalda Kia, Hasnah Osman, Vikneswaran al Murugaiyah, Madhukar Hemamalini, Hoong-Kun Fun

**Affiliations:** a School of Chemical Sciences, Universiti Sains Malaysia, 11800 USM, Penang, Malaysia; b School of Pharmaceutical Sciences, Universiti Sains Malaysia, 11800 USM, Penang, Malaysia; c X-ray Crystallography Unit, School of Physics, Universiti Sains Malaysia, 11800 USM, Penang, Malaysia

## Abstract

The title compound, C_9_H_11_N_3_O, was synthesized by the reaction of 4-methyl­benzaldehyde with semicarbazide. The mol­ecule adopts an *E* configuration about the central C=N double bond and the dihedral angle between the mean planes of the benzene ring and the carboxamide groups is 17.05 (9)°. The hydrazine N atoms are twisted slightly out of the plane of the carboxamide group [C—C—N—N torsion angle = 178.39 (14)°] and an intra­molecular N—H⋯N bond generates an *S*(5) ring. In the crystal, adjacent mol­ecules are connected *via* a pair of N—H⋯O hydrogen bonds, generating *R*_2_^2^(8) loops, resulting in supra­molecular [001] ribbons.

## Related literature

For applications of Schiff bases, see: Dhar *et al.* (1982 ▶); Przybylski *et al.* (2009 ▶); Bringmann *et al.* (2004 ▶); De Souza *et al.* (2007 ▶); Guo *et al.* (2007 ▶). For hydrogen-bond motifs, see: Bernstein *et al.* (1995 ▶).
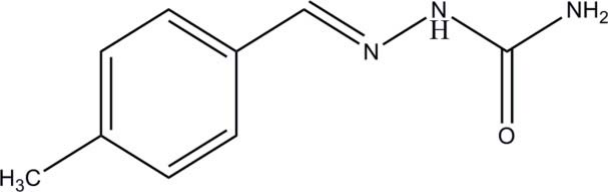


## Experimental

### 

#### Crystal data

C_9_H_11_N_3_O
*M* *_r_* = 177.21
Monoclinic, 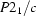

*a* = 17.2186 (13) Å
*b* = 4.5304 (3) Å
*c* = 11.9846 (9) Å
β = 93.348 (3)°
*V* = 933.29 (12) Å^3^
*Z* = 4
Mo *K*α radiation
μ = 0.09 mm^−1^
*T* = 296 K
0.76 × 0.23 × 0.05 mm

#### Data collection

Bruker SMART APEXII CCD diffractometer
Absorption correction: multi-scan (*SADABS*; Bruker, 2009 ▶) *T*_min_ = 0.937, *T*_max_ = 0.996
6322 measured reflections
1833 independent reflections
1285 reflections with *I* > 2σ(*I*)
*R*_int_ = 0.025

#### Refinement

*R*[*F* ^2^ > 2σ(*F* ^2^)] = 0.048
*wR*(*F* ^2^) = 0.149
*S* = 1.09
1833 reflections
131 parameters
H atoms treated by a mixture of independent and constrained refinement
Δρ_max_ = 0.18 e Å^−3^
Δρ_min_ = −0.18 e Å^−3^

### 

Data collection: *APEX2* (Bruker, 2009 ▶); cell refinement: *SAINT* (Bruker, 2009 ▶); data reduction: *SAINT*; program(s) used to solve structure: *SHELXTL* (Sheldrick, 2008 ▶); program(s) used to refine structure: *SHELXTL*; molecular graphics: *SHELXTL*; software used to prepare material for publication: *SHELXTL* and *PLATON* (Spek, 2009 ▶).

## Supplementary Material

Crystal structure: contains datablocks global, I. DOI: 10.1107/S1600536810052797/hb5772sup1.cif

Structure factors: contains datablocks I. DOI: 10.1107/S1600536810052797/hb5772Isup2.hkl

Additional supplementary materials:  crystallographic information; 3D view; checkCIF report

## Figures and Tables

**Table 1:** Hydrogen-bond geometry (Å, °)

*D*—H⋯*A*	*D*—H	H⋯*A*	*D*⋯*A*	*D*—H⋯*A*
N2—H1*N*2⋯O1^i^	0.928 (18)	1.998 (18)	2.9260 (19)	177.7 (17)
N3—H2*N*3⋯N1	0.93 (2)	2.22 (2)	2.667 (2)	108.6 (16)
N3—H1*N*3⋯O1^ii^	0.97 (2)	1.97 (2)	2.9106 (19)	163.5 (17)

Symmetry codes: (i) 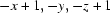; (ii) 
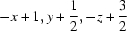
.

**Fig. 1.**
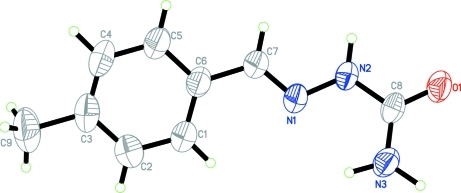
The asymmetric unit of the title compound, showing 50% probability displacement ellipsoids.

**Fig. 2.**
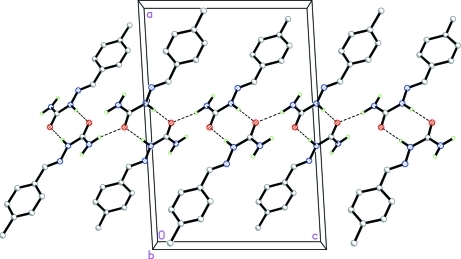
A supramolecular ribbon generated by N—H···O hydrogen bonds.

## supplementary crystallographic information

### Comment

Schiff bases are formed from the reaction of a primary amine with aldehydes or ketones. They exhibit interesting biological activities, such as antifungal, antibacterial, antimalarial, antiproliferative, anti-inflammatory, antiviral and antipyretic properties (Dhar *et al.*, 1982; Przybylski *et al.*, 2009). The Imine functional group present in these compounds is responsible for their vast biological activities. In addition, Schiff bases are also employed as intermediates in the total synthesis of bioactive natural products (Bringmann *et al.*, 2004; De Souza *et al.*, 2007; Guo *et al.*, 2007).

The asymmetric unit of the title compound is shown in Fig. 1.  The molecule adopts an *E* configuration about the central C═N double bond. The dihedral angle between the mean planes of the benzene (C1–C6) ring and carboxamide (N1–N3/O1/C8) group is 17.05 (9)°. The hydrazine N atoms are twisted slightly out of the plane of the carboxamide group [C6-C7-N1-N2 torsion angle = 178.39 (14)°].

In the crystal packing (Fig. 2), the adjacent molecules are connected via pair of N—H···O hydrogen bonds, generating an *R*_2_^2^(8) ring motifs, resulting in supramolecular ribbons along the *c*-axis.

### Experimental

A mixture of 4-methylbenzaldehyde (0.1 g, 0.83 mmol) and semicarbazide (0.062 g, 0.83 mmol) was dissolved in ethanol (5.0 ml)  and water (1.0 ml) which was then refluxed in the presence of sodium hydroxide (0.25M) for 3-4 hours. After completion of the reaction (through TLC monitoring), the mixture was poured into ice. The precipitate which was formed was filtered and washed with water. The pure solid was then recrystallised from ethanol to afford the title compound as colourless plates.

### Refinement

Atoms H1N2 and H1N3 were located from a difference Fourier map and refined freely [N–H = 0.93 (2)–0.97 (2) Å]. The remaining H atoms were positioned geometrically [C–H = 0.93–0.96 Å] and were refined using a riding model, with *U*_iso_(H) = 1.2 or 1.5 *U*_eq_(C).

### Crystal data

**Table:**

C_9_H_11_N_3_O	*F*(000) = 376
*M**_r_* = 177.21	*D*_x_ = 1.261 Mg m^−^^3^
Monoclinic, *P*2_1_/*c*	Mo *K*α radiation, λ = 0.71073 Å
Hall symbol: -P 2ybc	Cell parameters from 1533 reflections
*a* = 17.2186 (13) Å	θ = 3.4–22.6°
*b* = 4.5304 (3) Å	µ = 0.09 mm^−^^1^
*c* = 11.9846 (9) Å	*T* = 296 K
β = 93.348 (3)°	Plate, colourless
*V* = 933.29 (12) Å^3^	0.76 × 0.23 × 0.05 mm
*Z* = 4	

### Data collection

**Table:**

Bruker SMART APEXII CCD diffractometer	1833 independent reflections
Radiation source: fine-focus sealed tube	1285 reflections with *I* > 2σ(*I*)
graphite	*R*_int_ = 0.025
φ and ω scans	θ_max_ = 26.0°, θ_min_ = 2.4°
Absorption correction: multi-scan (*SADABS*; Bruker, 2009)	*h* = −21→20
*T*_min_ = 0.937, *T*_max_ = 0.996	*k* = −5→5
6322 measured reflections	*l* = −14→12

### Refinement

**Table:**

Refinement on *F*^2^	Primary atom site location: structure-invariant direct methods
Least-squares matrix: full	Secondary atom site location: difference Fourier map
*R*[*F*^2^ > 2σ(*F*^2^)] = 0.048	Hydrogen site location: inferred from neighbouring sites
*wR*(*F*^2^) = 0.149	H atoms treated by a mixture of independent and constrained refinement
*S* = 1.09	*w* = 1/[σ^2^(*F*_o_^2^) + (0.0825*P*)^2^ + 0.0212*P*] where *P* = (*F*_o_^2^ + 2*F*_c_^2^)/3
1833 reflections	(Δ/σ)_max_ < 0.001
131 parameters	Δρ_max_ = 0.18 e Å^−^^3^
0 restraints	Δρ_min_ = −0.18 e Å^−^^3^

### Special details

**Table:**

Geometry. All s.u.'s (except the s.u. in the dihedral angle between two l.s. planes) are estimated using the full covariance matrix. The cell s.u.'s are taken into account individually in the estimation of s.u.'s in distances, angles and torsion angles; correlations between s.u.'s in cell parameters are only used when they are defined by crystal symmetry. An approximate (isotropic) treatment of cell s.u.'s is used for estimating s.u.'s involving l.s. planes.
Refinement. Refinement of F^2^ against ALL reflections. The weighted R-factor wR and goodness of fit S are based on F^2^, conventional R-factors R are based on F, with F set to zero for negative F^2^. The threshold expression of F^2^ > 2σ(F^2^) is used only for calculating R-factors(gt) etc. and is not relevant to the choice of reflections for refinement. R-factors based on F^2^ are statistically about twice as large as those based on F, and R- factors based on ALL data will be even larger.

### Fractional atomic coordinates and isotropic or equivalent isotropic displacement parameters (Å^2^)

**Table:**

	*x*	*y*	*z*	*U*_iso_*/*U*_eq_	
O1	0.50913 (6)	0.2012 (3)	0.63337 (10)	0.0540 (4)	
N1	0.34815 (7)	0.3857 (3)	0.45522 (11)	0.0487 (4)	
N2	0.41216 (8)	0.2357 (3)	0.49998 (12)	0.0505 (4)	
H1N2	0.4366 (10)	0.093 (4)	0.4589 (16)	0.067 (6)*	
N3	0.42019 (9)	0.5689 (3)	0.64575 (12)	0.0530 (4)	
H2N3	0.3792 (10)	0.660 (5)	0.6044 (18)	0.076 (6)*	
H1N3	0.4490 (10)	0.643 (4)	0.7121 (19)	0.072 (6)*	
C1	0.20643 (10)	0.6318 (5)	0.35353 (17)	0.0676 (6)	
H1A	0.2179	0.6835	0.4278	0.081*	
C2	0.14128 (11)	0.7477 (5)	0.2966 (2)	0.0769 (7)	
H2A	0.1093	0.8755	0.3337	0.092*	
C3	0.12229 (11)	0.6785 (5)	0.18552 (18)	0.0663 (6)	
C4	0.17074 (11)	0.4889 (5)	0.13342 (15)	0.0671 (6)	
H4A	0.1595	0.4394	0.0589	0.081*	
C5	0.23599 (10)	0.3697 (5)	0.18930 (15)	0.0647 (6)	
H5A	0.2677	0.2417	0.1519	0.078*	
C6	0.25475 (9)	0.4387 (4)	0.30032 (13)	0.0520 (5)	
C7	0.32332 (10)	0.3054 (4)	0.35755 (14)	0.0531 (5)	
H7A	0.3497	0.1574	0.3216	0.064*	
C8	0.44997 (9)	0.3340 (4)	0.59601 (13)	0.0443 (4)	
C9	0.05146 (12)	0.8111 (6)	0.1244 (2)	0.0941 (8)	
H9A	0.0215	0.6574	0.0873	0.141*	
H9B	0.0676	0.9508	0.0702	0.141*	
H9C	0.0202	0.9092	0.1769	0.141*	

### Atomic displacement parameters (Å^2^)

**Table:**

	*U*^11^	*U*^22^	*U*^33^	*U*^12^	*U*^13^	*U*^23^
O1	0.0579 (7)	0.0596 (8)	0.0426 (7)	0.0045 (6)	−0.0143 (6)	0.0028 (5)
N1	0.0481 (8)	0.0571 (9)	0.0396 (8)	0.0016 (6)	−0.0072 (6)	0.0002 (6)
N2	0.0525 (8)	0.0584 (9)	0.0388 (8)	0.0084 (7)	−0.0125 (6)	−0.0024 (7)
N3	0.0623 (9)	0.0541 (9)	0.0410 (8)	0.0013 (7)	−0.0105 (7)	−0.0040 (7)
C1	0.0656 (11)	0.0847 (14)	0.0503 (11)	0.0149 (10)	−0.0135 (9)	−0.0105 (10)
C2	0.0643 (12)	0.0895 (15)	0.0749 (15)	0.0209 (11)	−0.0122 (11)	−0.0104 (12)
C3	0.0569 (11)	0.0752 (13)	0.0642 (12)	−0.0046 (10)	−0.0192 (10)	0.0128 (10)
C4	0.0674 (11)	0.0875 (14)	0.0443 (10)	−0.0055 (11)	−0.0160 (9)	0.0042 (10)
C5	0.0612 (11)	0.0872 (14)	0.0445 (10)	0.0076 (10)	−0.0080 (8)	−0.0041 (10)
C6	0.0497 (9)	0.0642 (11)	0.0412 (9)	−0.0002 (8)	−0.0056 (7)	0.0008 (8)
C7	0.0523 (9)	0.0656 (11)	0.0405 (9)	0.0088 (8)	−0.0050 (8)	−0.0040 (8)
C8	0.0491 (9)	0.0485 (10)	0.0343 (8)	−0.0080 (7)	−0.0058 (7)	0.0078 (7)
C9	0.0724 (14)	0.1056 (18)	0.100 (2)	0.0093 (13)	−0.0354 (13)	0.0148 (15)

### Geometric parameters (Å, °)

**Table:**

O1—C8	1.2436 (18)	C2—H2A	0.9300
N1—C7	1.275 (2)	C3—C4	1.373 (3)
N1—N2	1.3766 (18)	C3—C9	1.510 (2)
N2—C8	1.363 (2)	C4—C5	1.384 (2)
N2—H1N2	0.93 (2)	C4—H4A	0.9300
N3—C8	1.337 (2)	C5—C6	1.386 (2)
N3—H2N3	0.935 (19)	C5—H5A	0.9300
N3—H1N3	0.97 (2)	C6—C7	1.461 (2)
C1—C2	1.382 (2)	C7—H7A	0.9300
C1—C6	1.388 (3)	C9—H9A	0.9600
C1—H1A	0.9300	C9—H9B	0.9600
C2—C3	1.388 (3)	C9—H9C	0.9600
			
C7—N1—N2	115.78 (15)	C4—C5—C6	120.91 (19)
C8—N2—N1	119.98 (15)	C4—C5—H5A	119.5
C8—N2—H1N2	117.8 (11)	C6—C5—H5A	119.5
N1—N2—H1N2	120.9 (11)	C5—C6—C1	118.08 (16)
C8—N3—H2N3	114.4 (13)	C5—C6—C7	119.61 (17)
C8—N3—H1N3	116.6 (11)	C1—C6—C7	122.30 (15)
H2N3—N3—H1N3	127.9 (19)	N1—C7—C6	122.12 (16)
C2—C1—C6	120.24 (18)	N1—C7—H7A	118.9
C2—C1—H1A	119.9	C6—C7—H7A	118.9
C6—C1—H1A	119.9	O1—C8—N3	123.50 (15)
C1—C2—C3	121.8 (2)	O1—C8—N2	119.12 (16)
C1—C2—H2A	119.1	N3—C8—N2	117.37 (15)
C3—C2—H2A	119.1	C3—C9—H9A	109.5
C4—C3—C2	117.53 (17)	C3—C9—H9B	109.5
C4—C3—C9	121.5 (2)	H9A—C9—H9B	109.5
C2—C3—C9	120.9 (2)	C3—C9—H9C	109.5
C3—C4—C5	121.45 (18)	H9A—C9—H9C	109.5
C3—C4—H4A	119.3	H9B—C9—H9C	109.5
C5—C4—H4A	119.3		
			
C7—N1—N2—C8	170.10 (15)	C4—C5—C6—C7	178.99 (16)
C6—C1—C2—C3	−0.6 (3)	C2—C1—C6—C5	0.7 (3)
C1—C2—C3—C4	0.1 (3)	C2—C1—C6—C7	−178.66 (19)
C1—C2—C3—C9	−179.11 (19)	N2—N1—C7—C6	178.39 (14)
C2—C3—C4—C5	0.2 (3)	C5—C6—C7—N1	171.99 (17)
C9—C3—C4—C5	179.42 (19)	C1—C6—C7—N1	−8.7 (3)
C3—C4—C5—C6	−0.1 (3)	N1—N2—C8—O1	−177.76 (13)
C4—C5—C6—C1	−0.4 (3)	N1—N2—C8—N3	3.0 (2)

### Hydrogen-bond geometry (Å, °)

**Table:**

*D*—H···*A*	*D*—H	H···*A*	*D*···*A*	*D*—H···*A*
N2—H1N2···O1^i^	0.928 (18)	1.998 (18)	2.9260 (19)	177.7 (17)
N3—H2N3···N1	0.93 (2)	2.22 (2)	2.667 (2)	108.6 (16)
N3—H1N3···O1^ii^	0.97 (2)	1.97 (2)	2.9106 (19)	163.5 (17)

Symmetry codes: (i) −*x*+1, −*y*, −*z*+1; (ii) −*x*+1, *y*+1/2, −*z*+3/2.
